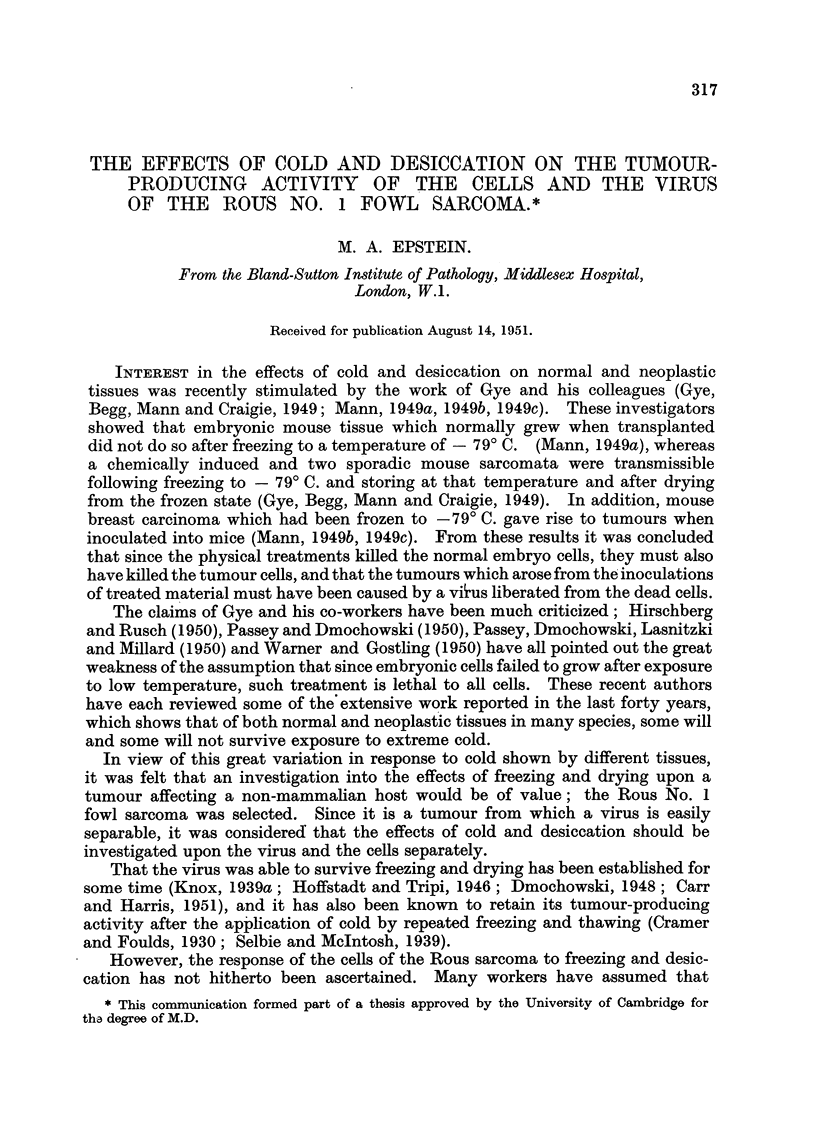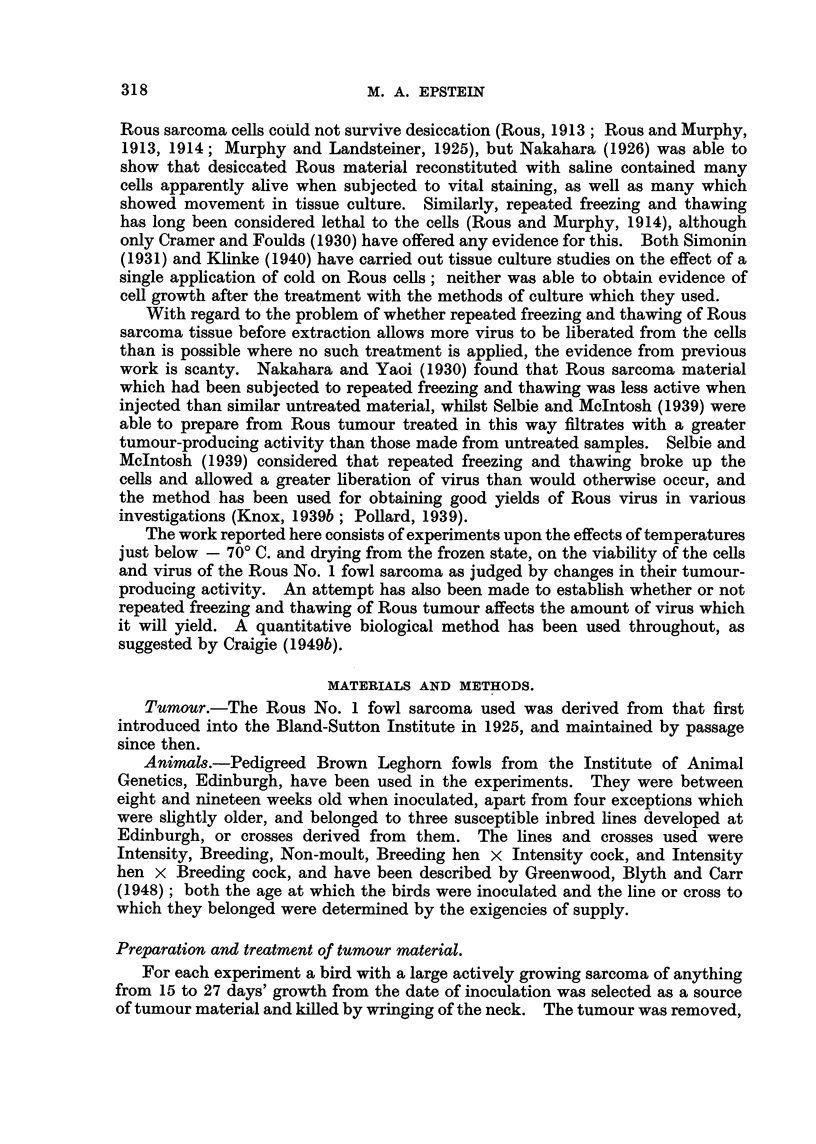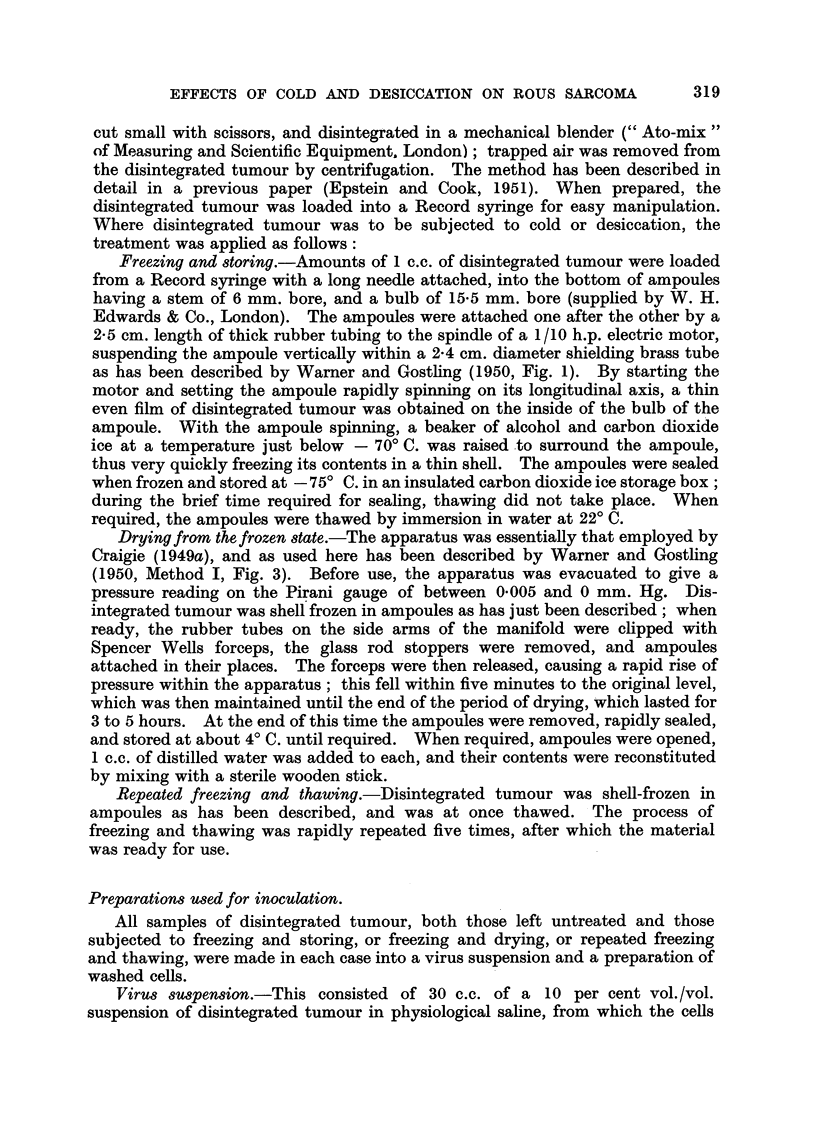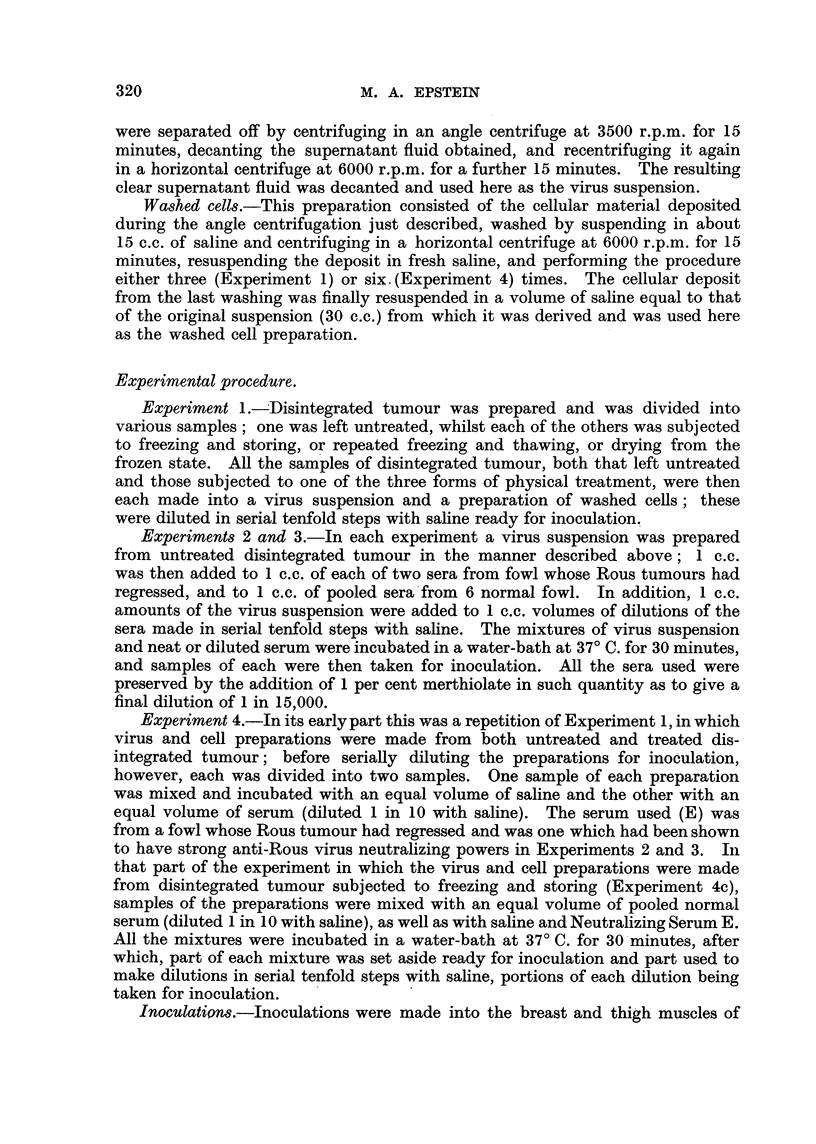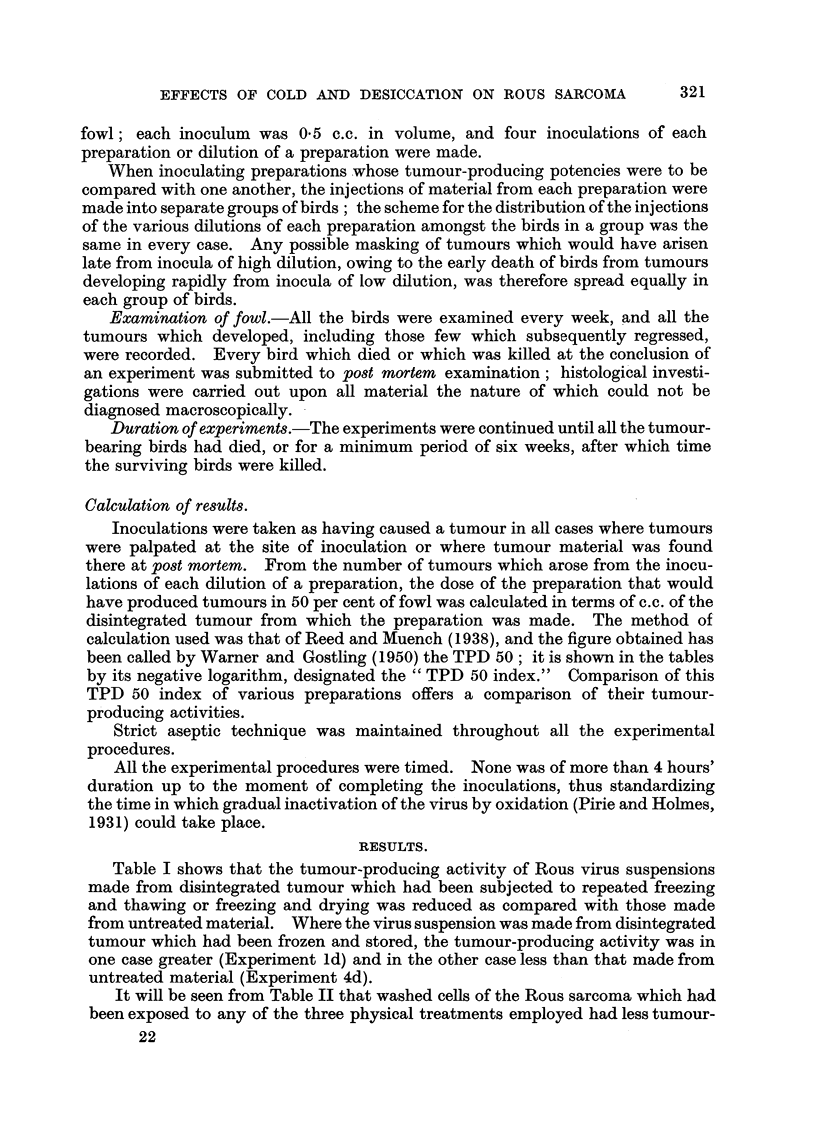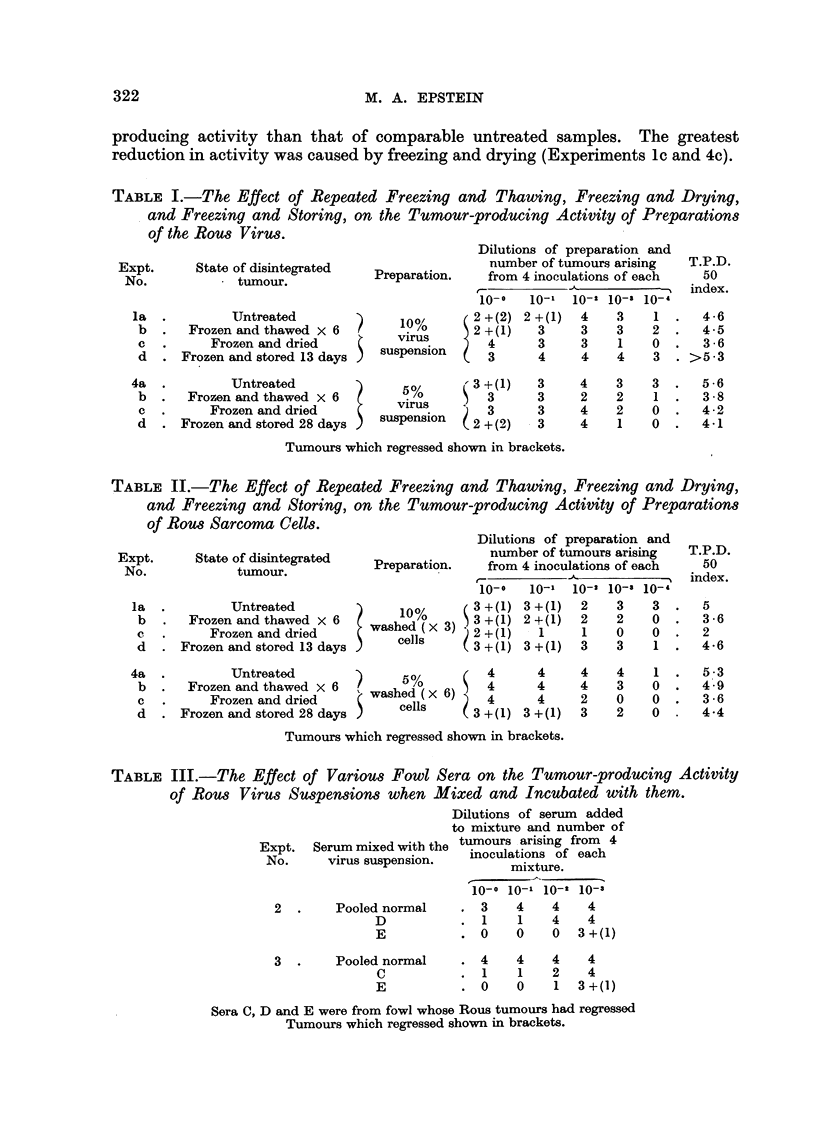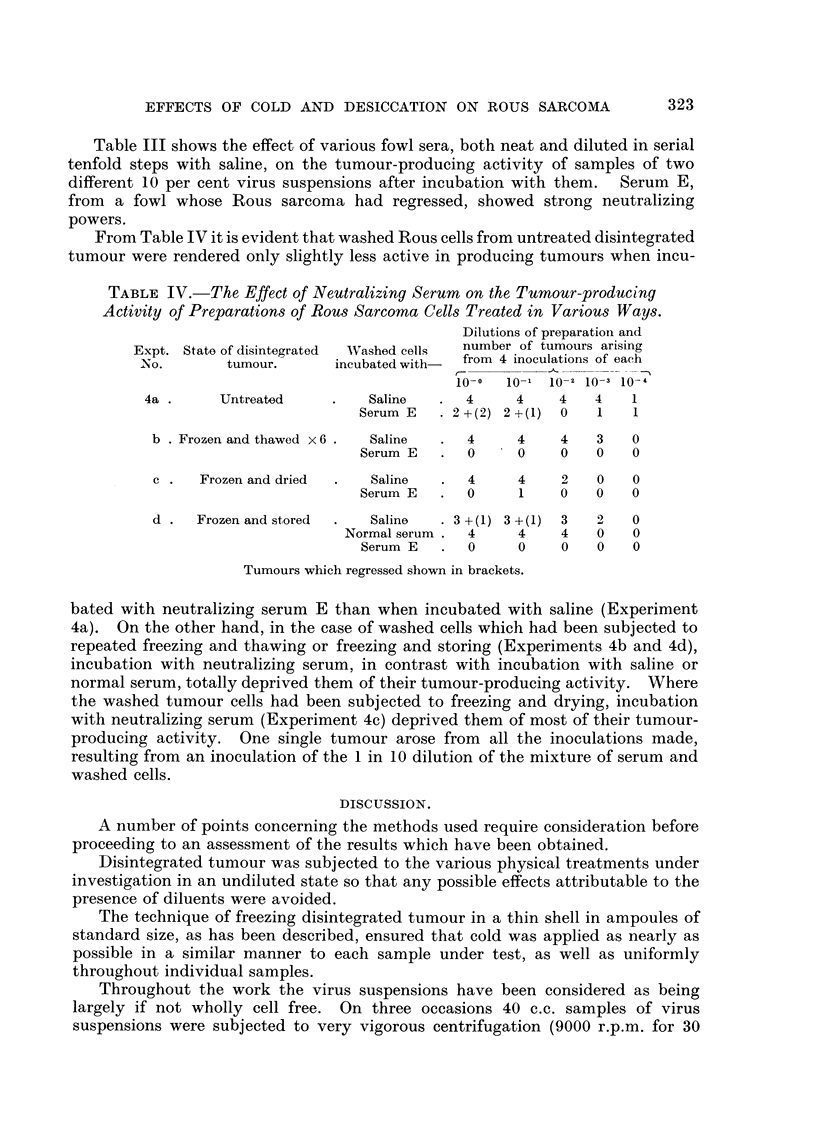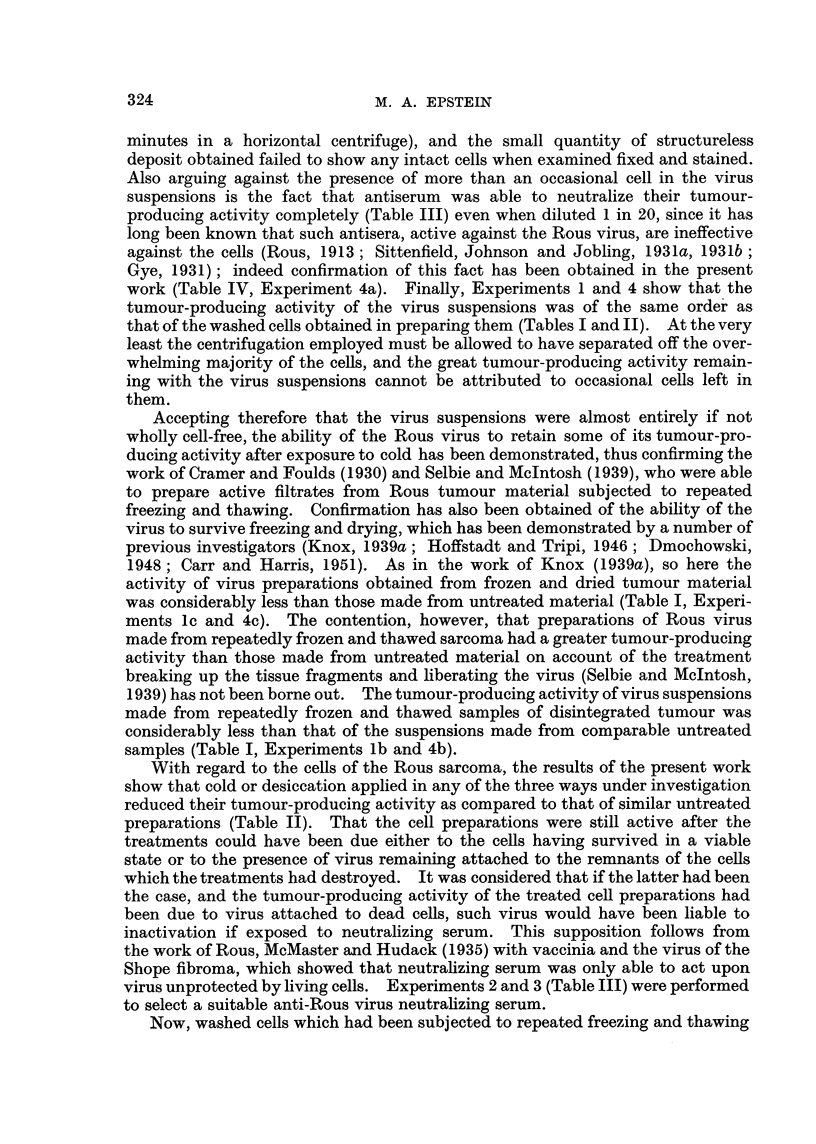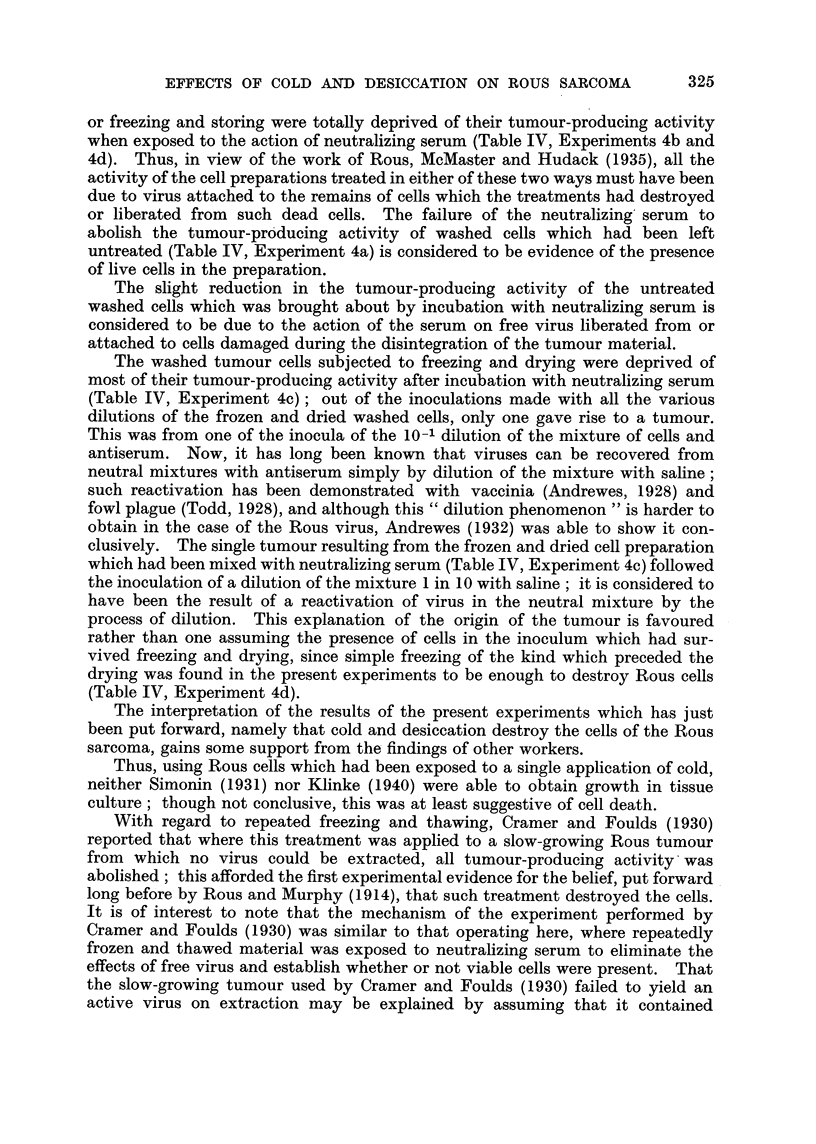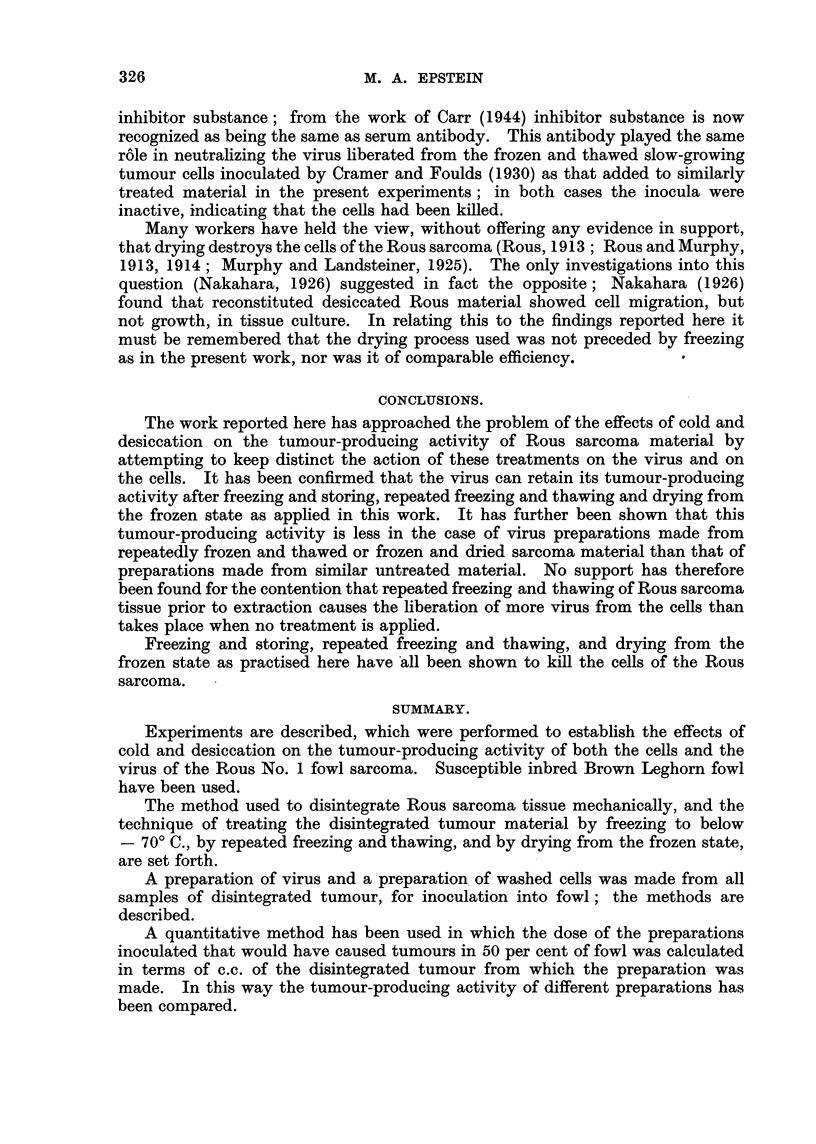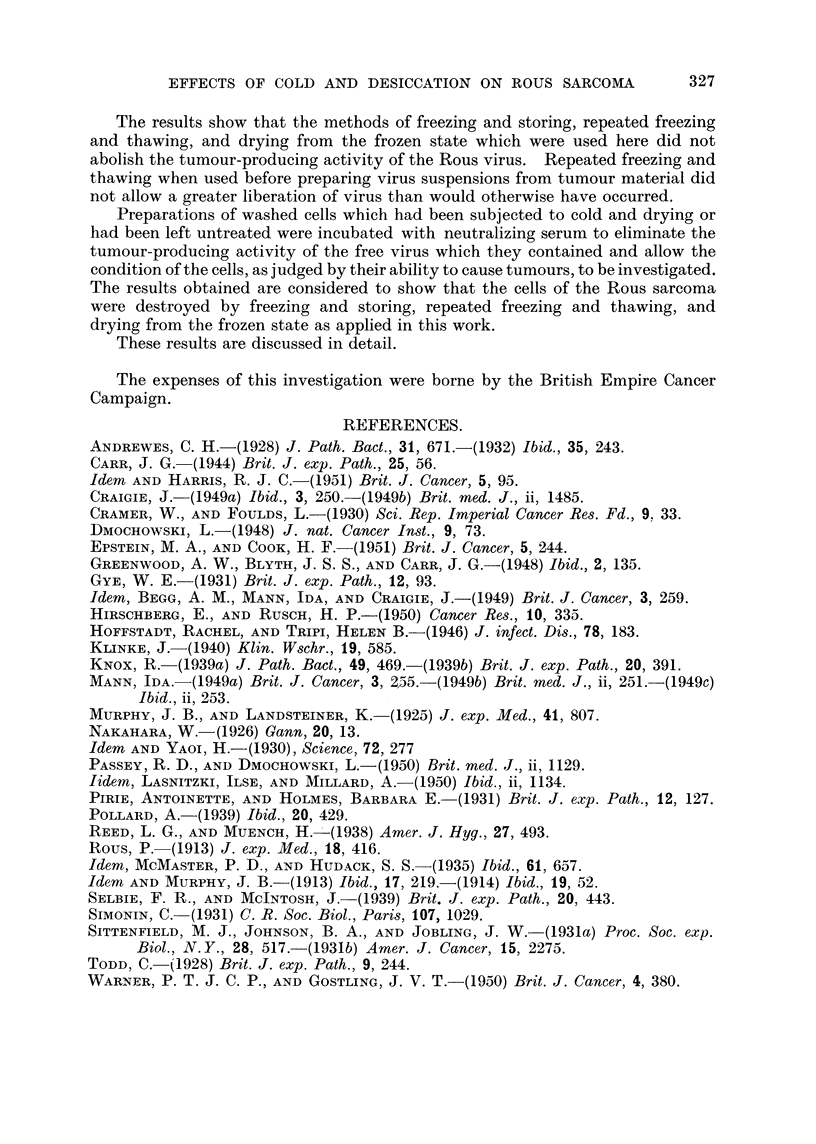# The Effects of Cold and Desiccation on the Tumour-Producing Activity of the Cells and the Virus of the Rous No. 1 Fowl Sarcoma*

**DOI:** 10.1038/bjc.1951.33

**Published:** 1951-09

**Authors:** M. A. Epstein


### THE EFFECTS OF COLD AND DESICCATION ON THE TUMOUR-PRODUCING ACTIVITY OF THE CELLS AND THE VIRUS OF THE ROUS NO. 1 FOWL SARCOMA.*

M. A. EPSTEIN.

*From the Bland-Sutton Institute of Pathology, Middlesex Hospital, London, W.1.*

Received for publication August 14, 1951.

INTEREST in the effects of cold and desiccation on normal and neoplastic tissues was recently stimulated by the work of Gye and his colleagues (Gye, Begg, Mann and Craigie, 1949; Mann, 1949*a*, 1949*b*, 1949*c*). These investigators showed that embryonic mouse tissue which normally grew when transplanted did not do so after freezing to a temperature of — 79° C. (Mann, 1949*a*), whereas a chemically induced and two sporadic mouse sarcomata were transmissible following freezing to — 79° C. and storing at that temperature and after drying from the frozen state (Gye, Begg, Mann and Craigie, 1949). In addition, mouse breast carcinoma which had been frozen to —79° C. gave rise to tumours when inoculated into mice (Mann, 1949*b*, 1949*c*). From these results it was concluded that since the physical treatments killed the normal embryo cells, they must also have killed the tumour cells, and that the tumours which arose from the inoculations of treated material must have been caused by a virus liberated from the dead cells.

The claims of Gye and his co-workers have been much criticized; Hirschberg and Rusch (1950), Passey and Dmochowski (1950), Passey, Dmochowski, Lasnitzki and Millard (1950) and Warner and Gostling (1950) have all pointed out the great weakness of the assumption that since embryonic cells failed to grow after exposure to low temperature, such treatment is lethal to all cells. These recent authors have each reviewed some of the extensive work reported in the last forty years, which shows that of both normal and neoplastic tissues in many species, some will and some will not survive exposure to extreme cold.

In view of this great variation in response to cold shown by different tissues, it was felt that an investigation into the effects of freezing and drying upon a tumour affecting a non-mammalian host would be of value; the Rous No. 1 fowl sarcoma was selected. Since it is a tumour from which a virus is easily separable, it was considered that the effects of cold and desiccation should be investigated upon the virus and the cells separately.

That the virus was able to survive freezing and drying has been established for some time (Knox, 1939*a*; Hoffstadt and Tripi, 1946; Dmochowski, 1948; Carr and Harris, 1951), and it has also been known to retain its tumour-producing activity after the application of cold by repeated freezing and thawing (Cramer and Foulds, 1930; Selbie and McIntosh, 1939).

However, the response of the cells of the Rous sarcoma to freezing and desiccation has not hitherto been ascertained. Many workers have assumed that

* This communication formed part of a thesis approved by the University of Cambridge for the degree of M.D.

Rous sarcoma cells could not survive desiccation (Rous, 1913 ; Rous and Murphy, 1913, 1914 ; Murphy and Landsteiner, 1925), but Nakahara (1926) was able to show that desiccated Rous material reconstituted with saline contained many cells apparently alive when subjected to vital staining, as well as many which showed movement in tissue culture. Similarly, repeated freezing and thawing has long been considered lethal to the cells (Rous and Murphy, 1914), although only Cramer and Foulds (1930) have offered any evidence for this. Both Simonin (1931) and Klinke (1940) have carried out tissue culture studies on the effect of a single application of cold on Rous cells ; neither was able to obtain evidence of cell growth after the treatment with the methods of culture which they used.

With regard to the problem of whether repeated freezing and thawing of Rous sarcoma tissue before extraction allows more virus to be liberated from the cells than is possible where no such treatment is applied, the evidence from previous work is scanty. Nakahara and Yaoi (1930) found that Rous sarcoma material which had been subjected to repeated freezing and thawing was less active when injected than similar untreated material, whilst Selbie and McIntosh (1939) were able to prepare from Rous tumour treated in this way filtrates with a greater tumour-producing activity than those made from untreated samples. Selbie and McIntosh (1939) considered that repeated freezing and thawing broke up the cells and allowed a greater liberation of virus than would otherwise occur, and the method has been used for obtaining good yields of Rous virus in various investigations (Knox, 1939*b* ; Pollard, 1939).

The work reported here consists of experiments upon the effects of temperatures just below − 70° C. and drying from the frozen state, on the viability of the cells and virus of the Rous No. 1 fowl sarcoma as judged by changes in their tumour-producing activity. An attempt has also been made to establish whether or not repeated freezing and thawing of Rous tumour affects the amount of virus which it will yield. A quantitative biological method has been used throughout, as suggested by Craigie (1949*b*).

### MATERIALS AND METHODS.

*Tumour.*—The Rous No. 1 fowl sarcoma used was derived from that first introduced into the Bland-Sutton Institute in 1925, and maintained by passage since then.

*Animals.*—Pedigreed Brown Leghorn fowls from the Institute of Animal Genetics, Edinburgh, have been used in the experiments. They were between eight and nineteen weeks old when inoculated, apart from four exceptions which were slightly older, and belonged to three susceptible inbred lines developed at Edinburgh, or crosses derived from them. The lines and crosses used were Intensity, Breeding, Non-moult, Breeding hen × Intensity cock, and Intensity hen × Breeding cock, and have been described by Greenwood, Blyth and Carr (1948) ; both the age at which the birds were inoculated and the line or cross to which they belonged were determined by the exigencies of supply.

### *Preparation and treatment of tumour material.*

For each experiment a bird with a large actively growing sarcoma of anything from 15 to 27 days' growth from the date of inoculation was selected as a source of tumour material and killed by wringing of the neck. The tumour was removed, cut small with scissors, and disintegrated in a mechanical blender (" Ato-mix " of Measuring and Scientific Equipment, London) ; trapped air was removed from the disintegrated tumour by centrifugation. The method has been described in detail in a previous paper (Epstein and Cook, 1951). When prepared, the disintegrated tumour was loaded into a Record syringe for easy manipulation. Where disintegrated tumour was to be subjected to cold or desiccation, the treatment was applied as follows :

*Freezing and storing.*—Amounts of 1 c.c. of disintegrated tumour were loaded from a Record syringe with a long needle attached, into the bottom of ampoules having a stem of 6 mm. bore, and a bulb of 15·5 mm. bore (supplied by W. H. Edwards & Co., London). The ampoules were attached one after the other by a 2·5 cm. length of thick rubber tubing to the spindle of a 1/10 h.p. electric motor, suspending the ampoule vertically within a 2·4 cm. diameter shielding brass tube as has been described by Warner and Gostling (1950, Fig. 1). By starting the motor and setting the ampoule rapidly spinning on its longitudinal axis, a thin even film of disintegrated tumour was obtained on the inside of the bulb of the ampoule. With the ampoule spinning, a beaker of alcohol and carbon dioxide ice at a temperature just below $-70°$ C. was raised to surround the ampoule, thus very quickly freezing its contents in a thin shell. The ampoules were sealed when frozen and stored at $-75°$ C. in an insulated carbon dioxide ice storage box ; during the brief time required for sealing, thawing did not take place. When required, the ampoules were thawed by immersion in water at 22° C.

*Drying from the frozen state.*—The apparatus was essentially that employed by Craigie (1949*a*), and as used here has been described by Warner and Gostling (1950, Method I, Fig. 3). Before use, the apparatus was evacuated to give a pressure reading on the Pirani gauge of between 0·005 and 0 mm. Hg. Disintegrated tumour was shell frozen in ampoules as has just been described ; when ready, the rubber tubes on the side arms of the manifold were clipped with Spencer Wells forceps, the glass rod stoppers were removed, and ampoules attached in their places. The forceps were then released, causing a rapid rise of pressure within the apparatus ; this fell within five minutes to the original level, which was then maintained until the end of the period of drying, which lasted for 3 to 5 hours. At the end of this time the ampoules were removed, rapidly sealed, and stored at about 4° C. until required. When required, ampoules were opened, 1 c.c. of distilled water was added to each, and their contents were reconstituted by mixing with a sterile wooden stick.

*Repeated freezing and thawing.*—Disintegrated tumour was shell-frozen in ampoules as has been described, and was at once thawed. The process of freezing and thawing was rapidly repeated five times, after which the material was ready for use.

*Preparations used for inoculation.*

All samples of disintegrated tumour, both those left untreated and those subjected to freezing and storing, or freezing and drying, or repeated freezing and thawing, were made in each case into a virus suspension and a preparation of washed cells.

*Virus suspension.*—This consisted of 30 c.c. of a 10 per cent vol./vol. suspension of disintegrated tumour in physiological saline, from which the cells were separated off by centrifuging in an angle centrifuge at 3500 r.p.m. for 15 minutes, decanting the supernatant fluid obtained, and recentrifuging it again in a horizontal centrifuge at 6000 r.p.m. for a further 15 minutes. The resulting clear supernatant fluid was decanted and used here as the virus suspension.

*Washed cells.*—This preparation consisted of the cellular material deposited during the angle centrifugation just described, washed by suspending in about 15 c.c. of saline and centrifuging in a horizontal centrifuge at 6000 r.p.m. for 15 minutes, resuspending the deposit in fresh saline, and performing the procedure either three (Experiment 1) or six (Experiment 4) times. The cellular deposit from the last washing was finally resuspended in a volume of saline equal to that of the original suspension (30 c.c.) from which it was derived and was used here as the washed cell preparation.

*Experimental procedure.*

*Experiment* 1.—Disintegrated tumour was prepared and was divided into various samples ; one was left untreated, whilst each of the others was subjected to freezing and storing, or repeated freezing and thawing, or drying from the frozen state. All the samples of disintegrated tumour, both that left untreated and those subjected to one of the three forms of physical treatment, were then each made into a virus suspension and a preparation of washed cells ; these were diluted in serial tenfold steps with saline ready for inoculation.

*Experiments* 2 *and* 3.—In each experiment a virus suspension was prepared from untreated disintegrated tumour in the manner described above ; 1 c.c. was then added to 1 c.c. of each of two sera from fowl whose Rous tumours had regressed, and to 1 c.c. of pooled sera from 6 normal fowl. In addition, 1 c.c. amounts of the virus suspension were added to 1 c.c. volumes of dilutions of the sera made in serial tenfold steps with saline. The mixtures of virus suspension and neat or diluted serum were incubated in a water-bath at 37° C. for 30 minutes, and samples of each were then taken for inoculation. All the sera used were preserved by the addition of 1 per cent merthiolate in such quantity as to give a final dilution of 1 in 15,000.

*Experiment* 4.—In its early part this was a repetition of Experiment 1, in which virus and cell preparations were made from both untreated and treated disintegrated tumour; before serially diluting the preparations for inoculation, however, each was divided into two samples. One sample of each preparation was mixed and incubated with an equal volume of saline and the other with an equal volume of serum (diluted 1 in 10 with saline). The serum used (E) was from a fowl whose Rous tumour had regressed and was one which had been shown to have strong anti-Rous virus neutralizing powers in Experiments 2 and 3. In that part of the experiment in which the virus and cell preparations were made from disintegrated tumour subjected to freezing and storing (Experiment 4c), samples of the preparations were mixed with an equal volume of pooled normal serum (diluted 1 in 10 with saline), as well as with saline and Neutralizing Serum E. All the mixtures were incubated in a water-bath at 37° C. for 30 minutes, after which, part of each mixture was set aside ready for inoculation and part used to make dilutions in serial tenfold steps with saline, portions of each dilution being taken for inoculation.

*Inoculations.*—Inoculations were made into the breast and thigh muscles of fowl; each inoculum was 0·5 c.c. in volume, and four inoculations of each preparation or dilution of a preparation were made.

When inoculating preparations whose tumour-producing potencies were to be compared with one another, the injections of material from each preparation were made into separate groups of birds; the scheme for the distribution of the injections of the various dilutions of each preparation amongst the birds in a group was the same in every case. Any possible masking of tumours which would have arisen late from inocula of high dilution, owing to the early death of birds from tumours developing rapidly from inocula of low dilution, was therefore spread equally in each group of birds.

*Examination of fowl.*—All the birds were examined every week, and all the tumours which developed, including those few which subsequently regressed, were recorded. Every bird which died or which was killed at the conclusion of an experiment was submitted to *post mortem* examination; histological investigations were carried out upon all material the nature of which could not be diagnosed macroscopically.

*Duration of experiments.*—The experiments were continued until all the tumour-bearing birds had died, or for a minimum period of six weeks, after which time the surviving birds were killed.

*Calculation of results.*

Inoculations were taken as having caused a tumour in all cases where tumours were palpated at the site of inoculation or where tumour material was found there at *post mortem*. From the number of tumours which arose from the inoculations of each dilution of a preparation, the dose of the preparation that would have produced tumours in 50 per cent of fowl was calculated in terms of c.c. of the disintegrated tumour from which the preparation was made. The method of calculation used was that of Reed and Muench (1938), and the figure obtained has been called by Warner and Gostling (1950) the TPD 50; it is shown in the tables by its negative logarithm, designated the "TPD 50 index." Comparison of this TPD 50 index of various preparations offers a comparison of their tumour-producing activities.

Strict aseptic technique was maintained throughout all the experimental procedures.

All the experimental procedures were timed. None was of more than 4 hours' duration up to the moment of completing the inoculations, thus standardizing the time in which gradual inactivation of the virus by oxidation (Pirie and Holmes, 1931) could take place.

RESULTS.

Table I shows that the tumour-producing activity of Rous virus suspensions made from disintegrated tumour which had been subjected to repeated freezing and thawing or freezing and drying was reduced as compared with those made from untreated material. Where the virus suspension was made from disintegrated tumour which had been frozen and stored, the tumour-producing activity was in one case greater (Experiment 1d) and in the other case less than that made from untreated material (Experiment 4d).

It will be seen from Table II that washed cells of the Rous sarcoma which had been exposed to any of the three physical treatments employed had less tumourproducing activity than that of comparable untreated samples. The greatest reduction in activity was caused by freezing and drying (Experiments 1c and 4c).

TABLE I.—*The Effect of Repeated Freezing and Thawing, Freezing and Drying, and Freezing and Storing, on the Tumour-producing Activity of Preparations of the Rous Virus.*

| Expt. No. | State of disintegrated tumour. | Preparation. | Dilutions of preparation and number of tumours arising from 4 inoculations of each: $10^{-0}$ | $10^{-1}$ | $10^{-2}$ | $10^{-3}$ | $10^{-4}$ | T.P.D. 50 index. |
|---|---|---|---|---|---|---|---|---|
| 1a | Untreated | 10% virus suspension | 2 +(2) | 2 +(1) | 4 | 3 | 1 | 4·6 |
| b | Frozen and thawed × 6 | | 2 +(1) | 3 | 3 | 3 | 2 | 4·5 |
| c | Frozen and dried | | 4 | 3 | 3 | 1 | 0 | 3·6 |
| d | Frozen and stored 13 days | | 3 | 4 | 4 | 4 | 3 | >5·3 |
| 4a | Untreated | 5% virus suspension | 3 +(1) | 3 | 4 | 3 | 3 | 5·6 |
| b | Frozen and thawed × 6 | | 3 | 3 | 2 | 2 | 1 | 3·8 |
| c | Frozen and dried | | 3 | 3 | 4 | 2 | 0 | 4·2 |
| d | Frozen and stored 28 days | | 2 +(2) | 3 | 4 | 1 | 0 | 4·1 |

Tumours which regressed shown in brackets.

TABLE II.—*The Effect of Repeated Freezing and Thawing, Freezing and Drying, and Freezing and Storing, on the Tumour-producing Activity of Preparations of Rous Sarcoma Cells.*

| Expt. No. | State of disintegrated tumour. | Preparation. | Dilutions of preparation and number of tumours arising from 4 inoculations of each: $10^{-0}$ | $10^{-1}$ | $10^{-2}$ | $10^{-3}$ | $10^{-4}$ | T.P.D. 50 index. |
|---|---|---|---|---|---|---|---|---|
| 1a | Untreated | 10% washed (× 3) cells | 3 +(1) | 3 +(1) | 2 | 3 | 3 | 5 |
| b | Frozen and thawed × 6 | | 3 +(1) | 2 +(1) | 2 | 2 | 0 | 3·6 |
| c | Frozen and dried | | 2 +(1) | 1 | 1 | 0 | 0 | 2 |
| d | Frozen and stored 13 days | | 3 +(1) | 3 +(1) | 3 | 3 | 1 | 4·6 |
| 4a | Untreated | 5% washed (× 6) cells | 4 | 4 | 4 | 4 | 1 | 5·3 |
| b | Frozen and thawed × 6 | | 4 | 4 | 4 | 3 | 0 | 4·9 |
| c | Frozen and dried | | 4 | 4 | 2 | 0 | 0 | 3·6 |
| d | Frozen and stored 28 days | | 3 +(1) | 3 +(1) | 3 | 2 | 0 | 4·4 |

Tumours which regressed shown in brackets.

TABLE III.—*The Effect of Various Fowl Sera on the Tumour-producing Activity of Rous Virus Suspensions when Mixed and Incubated with them.*

| Expt. No. | Serum mixed with the virus suspension. | Dilutions of serum added to mixture and number of tumours arising from 4 inoculations of each mixture: $10^{-0}$ | $10^{-1}$ | $10^{-2}$ | $10^{-3}$ |
|---|---|---|---|---|---|
| 2 | Pooled normal | 3 | 4 | 4 | 4 |
| | D | 1 | 1 | 4 | 4 |
| | E | 0 | 0 | 0 | 3 +(1) |
| 3 | Pooled normal | 4 | 4 | 4 | 4 |
| | C | 1 | 1 | 2 | 4 |
| | E | 0 | 0 | 1 | 3 +(1) |

Sera C, D and E were from fowl whose Rous tumours had regressed
Tumours which regressed shown in brackets.

Table III shows the effect of various fowl sera, both neat and diluted in serial tenfold steps with saline, on the tumour-producing activity of samples of two different 10 per cent virus suspensions after incubation with them. Serum E, from a fowl whose Rous sarcoma had regressed, showed strong neutralizing powers.

From Table IV it is evident that washed Rous cells from untreated disintegrated tumour were rendered only slightly less active in producing tumours when incubated with neutralizing serum E than when incubated with saline (Experiment 4a). On the other hand, in the case of washed cells which had been subjected to repeated freezing and thawing or freezing and storing (Experiments 4b and 4d), incubation with neutralizing serum, in contrast with incubation with saline or normal serum, totally deprived them of their tumour-producing activity. Where the washed tumour cells had been subjected to freezing and drying, incubation with neutralizing serum (Experiment 4c) deprived them of most of their tumour-producing activity. One single tumour arose from all the inoculations made, resulting from an inoculation of the 1 in 10 dilution of the mixture of serum and washed cells.

TABLE IV.—*The Effect of Neutralizing Serum on the Tumour-producing Activity of Preparations of Rous Sarcoma Cells Treated in Various Ways.*

| Expt. No. | State of disintegrated tumour. | Washed cells incubated with— | Dilutions of preparation and number of tumours arising from 4 inoculations of each | | | | |
|---|---|---|---|---|---|---|---|
| | | | $10^{-0}$ | $10^{-1}$ | $10^{-2}$ | $10^{-3}$ | $10^{-4}$ |
| 4a | Untreated | Saline | 4 | 4 | 4 | 4 | 1 |
| | | Serum E | 2 +(2) | 2 +(1) | 0 | 1 | 1 |
| b | Frozen and thawed ×6 | Saline | 4 | 4 | 4 | 3 | 0 |
| | | Serum E | 0 | 0 | 0 | 0 | 0 |
| c | Frozen and dried | Saline | 4 | 4 | 2 | 0 | 0 |
| | | Serum E | 0 | 1 | 0 | 0 | 0 |
| d | Frozen and stored | Saline | 3 +(1) | 3 +(1) | 3 | 2 | 0 |
| | | Normal serum | 4 | 4 | 4 | 0 | 0 |
| | | Serum E | 0 | 0 | 0 | 0 | 0 |

Tumours which regressed shown in brackets.

### DISCUSSION.

A number of points concerning the methods used require consideration before proceeding to an assessment of the results which have been obtained.

Disintegrated tumour was subjected to the various physical treatments under investigation in an undiluted state so that any possible effects attributable to the presence of diluents were avoided.

The technique of freezing disintegrated tumour in a thin shell in ampoules of standard size, as has been described, ensured that cold was applied as nearly as possible in a similar manner to each sample under test, as well as uniformly throughout individual samples.

Throughout the work the virus suspensions have been considered as being largely if not wholly cell free. On three occasions 40 c.c. samples of virus suspensions were subjected to very vigorous centrifugation (9000 r.p.m. for 30

minutes in a horizontal centrifuge), and the small quantity of structureless deposit obtained failed to show any intact cells when examined fixed and stained. Also arguing against the presence of more than an occasional cell in the virus suspensions is the fact that antiserum was able to neutralize their tumour-producing activity completely (Table III) even when diluted 1 in 20, since it has long been known that such antisera, active against the Rous virus, are ineffective against the cells (Rous, 1913; Sittenfield, Johnson and Jobling, 1931*a*, 1931*b*; Gye, 1931); indeed confirmation of this fact has been obtained in the present work (Table IV, Experiment 4a). Finally, Experiments 1 and 4 show that the tumour-producing activity of the virus suspensions was of the same order as that of the washed cells obtained in preparing them (Tables I and II). At the very least the centrifugation employed must be allowed to have separated off the overwhelming majority of the cells, and the great tumour-producing activity remaining with the virus suspensions cannot be attributed to occasional cells left in them.

Accepting therefore that the virus suspensions were almost entirely if not wholly cell-free, the ability of the Rous virus to retain some of its tumour-producing activity after exposure to cold has been demonstrated, thus confirming the work of Cramer and Foulds (1930) and Selbie and McIntosh (1939), who were able to prepare active filtrates from Rous tumour material subjected to repeated freezing and thawing. Confirmation has also been obtained of the ability of the virus to survive freezing and drying, which has been demonstrated by a number of previous investigators (Knox, 1939*a*; Hoffstadt and Tripi, 1946; Dmochowski, 1948; Carr and Harris, 1951). As in the work of Knox (1939*a*), so here the activity of virus preparations obtained from frozen and dried tumour material was considerably less than those made from untreated material (Table I, Experiments 1c and 4c). The contention, however, that preparations of Rous virus made from repeatedly frozen and thawed sarcoma had a greater tumour-producing activity than those made from untreated material on account of the treatment breaking up the tissue fragments and liberating the virus (Selbie and McIntosh, 1939) has not been borne out. The tumour-producing activity of virus suspensions made from repeatedly frozen and thawed samples of disintegrated tumour was considerably less than that of the suspensions made from comparable untreated samples (Table I, Experiments 1b and 4b).

With regard to the cells of the Rous sarcoma, the results of the present work show that cold or desiccation applied in any of the three ways under investigation reduced their tumour-producing activity as compared to that of similar untreated preparations (Table II). That the cell preparations were still active after the treatments could have been due either to the cells having survived in a viable state or to the presence of virus remaining attached to the remnants of the cells which the treatments had destroyed. It was considered that if the latter had been the case, and the tumour-producing activity of the treated cell preparations had been due to virus attached to dead cells, such virus would have been liable to inactivation if exposed to neutralizing serum. This supposition follows from the work of Rous, McMaster and Hudack (1935) with vaccinia and the virus of the Shope fibroma, which showed that neutralizing serum was only able to act upon virus unprotected by living cells. Experiments 2 and 3 (Table III) were performed to select a suitable anti-Rous virus neutralizing serum.

Now, washed cells which had been subjected to repeated freezing and thawing or freezing and storing were totally deprived of their tumour-producing activity when exposed to the action of neutralizing serum (Table IV, Experiments 4b and 4d). Thus, in view of the work of Rous, McMaster and Hudack (1935), all the activity of the cell preparations treated in either of these two ways must have been due to virus attached to the remains of cells which the treatments had destroyed or liberated from such dead cells. The failure of the neutralizing serum to abolish the tumour-producing activity of washed cells which had been left untreated (Table IV, Experiment 4a) is considered to be evidence of the presence of live cells in the preparation.

The slight reduction in the tumour-producing activity of the untreated washed cells which was brought about by incubation with neutralizing serum is considered to be due to the action of the serum on free virus liberated from or attached to cells damaged during the disintegration of the tumour material.

The washed tumour cells subjected to freezing and drying were deprived of most of their tumour-producing activity after incubation with neutralizing serum (Table IV, Experiment 4c); out of the inoculations made with all the various dilutions of the frozen and dried washed cells, only one gave rise to a tumour. This was from one of the inocula of the $10^{-1}$ dilution of the mixture of cells and antiserum. Now, it has long been known that viruses can be recovered from neutral mixtures with antiserum simply by dilution of the mixture with saline; such reactivation has been demonstrated with vaccinia (Andrewes, 1928) and fowl plague (Todd, 1928), and although this "dilution phenomenon" is harder to obtain in the case of the Rous virus, Andrewes (1932) was able to show it conclusively. The single tumour resulting from the frozen and dried cell preparation which had been mixed with neutralizing serum (Table IV, Experiment 4c) followed the inoculation of a dilution of the mixture 1 in 10 with saline; it is considered to have been the result of a reactivation of virus in the neutral mixture by the process of dilution. This explanation of the origin of the tumour is favoured rather than one assuming the presence of cells in the inoculum which had survived freezing and drying, since simple freezing of the kind which preceded the drying was found in the present experiments to be enough to destroy Rous cells (Table IV, Experiment 4d).

The interpretation of the results of the present experiments which has just been put forward, namely that cold and desiccation destroy the cells of the Rous sarcoma, gains some support from the findings of other workers.

Thus, using Rous cells which had been exposed to a single application of cold, neither Simonin (1931) nor Klinke (1940) were able to obtain growth in tissue culture; though not conclusive, this was at least suggestive of cell death.

With regard to repeated freezing and thawing, Cramer and Foulds (1930) reported that where this treatment was applied to a slow-growing Rous tumour from which no virus could be extracted, all tumour-producing activity was abolished; this afforded the first experimental evidence for the belief, put forward long before by Rous and Murphy (1914), that such treatment destroyed the cells. It is of interest to note that the mechanism of the experiment performed by Cramer and Foulds (1930) was similar to that operating here, where repeatedly frozen and thawed material was exposed to neutralizing serum to eliminate the effects of free virus and establish whether or not viable cells were present. That the slow-growing tumour used by Cramer and Foulds (1930) failed to yield an active virus on extraction may be explained by assuming that it contained inhibitor substance; from the work of Carr (1944) inhibitor substance is now recognized as being the same as serum antibody. This antibody played the same rôle in neutralizing the virus liberated from the frozen and thawed slow-growing tumour cells inoculated by Cramer and Foulds (1930) as that added to similarly treated material in the present experiments; in both cases the inocula were inactive, indicating that the cells had been killed.

Many workers have held the view, without offering any evidence in support, that drying destroys the cells of the Rous sarcoma (Rous, 1913; Rous and Murphy, 1913, 1914; Murphy and Landsteiner, 1925). The only investigations into this question (Nakahara, 1926) suggested in fact the opposite; Nakahara (1926) found that reconstituted desiccated Rous material showed cell migration, but not growth, in tissue culture. In relating this to the findings reported here it must be remembered that the drying process used was not preceded by freezing as in the present work, nor was it of comparable efficiency.

### CONCLUSIONS.

The work reported here has approached the problem of the effects of cold and desiccation on the tumour-producing activity of Rous sarcoma material by attempting to keep distinct the action of these treatments on the virus and on the cells. It has been confirmed that the virus can retain its tumour-producing activity after freezing and storing, repeated freezing and thawing and drying from the frozen state as applied in this work. It has further been shown that this tumour-producing activity is less in the case of virus preparations made from repeatedly frozen and thawed or frozen and dried sarcoma material than that of preparations made from similar untreated material. No support has therefore been found for the contention that repeated freezing and thawing of Rous sarcoma tissue prior to extraction causes the liberation of more virus from the cells than takes place when no treatment is applied.

Freezing and storing, repeated freezing and thawing, and drying from the frozen state as practised here have all been shown to kill the cells of the Rous sarcoma.

### SUMMARY.

Experiments are described, which were performed to establish the effects of cold and desiccation on the tumour-producing activity of both the cells and the virus of the Rous No. 1 fowl sarcoma. Susceptible inbred Brown Leghorn fowl have been used.

The method used to disintegrate Rous sarcoma tissue mechanically, and the technique of treating the disintegrated tumour material by freezing to below − 70° C., by repeated freezing and thawing, and by drying from the frozen state, are set forth.

A preparation of virus and a preparation of washed cells was made from all samples of disintegrated tumour, for inoculation into fowl; the methods are described.

A quantitative method has been used in which the dose of the preparations inoculated that would have caused tumours in 50 per cent of fowl was calculated in terms of c.c. of the disintegrated tumour from which the preparation was made. In this way the tumour-producing activity of different preparations has been compared.

The results show that the methods of freezing and storing, repeated freezing and thawing, and drying from the frozen state which were used here did not abolish the tumour-producing activity of the Rous virus. Repeated freezing and thawing when used before preparing virus suspensions from tumour material did not allow a greater liberation of virus than would otherwise have occurred.

Preparations of washed cells which had been subjected to cold and drying or had been left untreated were incubated with neutralizing serum to eliminate the tumour-producing activity of the free virus which they contained and allow the condition of the cells, as judged by their ability to cause tumours, to be investigated. The results obtained are considered to show that the cells of the Rous sarcoma were destroyed by freezing and storing, repeated freezing and thawing, and drying from the frozen state as applied in this work.

These results are discussed in detail.

The expenses of this investigation were borne by the British Empire Cancer Campaign.

### REFERENCES.

ANDREWES, C. H.—(1928) *J. Path. Bact.*, **31**, 671.—(1932) *Ibid.*, **35**, 243.
CARR, J. G.—(1944) *Brit. J. exp. Path.*, **25**, 56.
*Idem* AND HARRIS, R. J. C.—(1951) *Brit. J. Cancer*, **5**, 95.
CRAIGIE, J.—(1949*a*) *Ibid.*, **3**, 250.—(1949*b*) *Brit. med. J.*, ii, 1485.
CRAMER, W., AND FOULDS, L.—(1930) *Sci. Rep. Imperial Cancer Res. Fd.*, **9**, 33.
DMOCHOWSKI, L.—(1948) *J. nat. Cancer Inst.*, **9**, 73.
EPSTEIN, M. A., AND COOK, H. F.—(1951) *Brit. J. Cancer*, **5**, 244.
GREENWOOD, A. W., BLYTH, J. S. S., AND CARR, J. G.—(1948) *Ibid.*, **2**, 135.
GYE, W. E.—(1931) *Brit. J. exp. Path.*, **12**, 93.
*Idem*, BEGG, A. M., MANN, IDA, AND CRAIGIE, J.—(1949) *Brit. J. Cancer*, **3**, 259.
HIRSCHBERG, E., AND RUSCH, H. P.—(1950) *Cancer Res.*, **10**, 335.
HOFFSTADT, RACHEL, AND TRIPI, HELEN B.—(1946) *J. infect. Dis.*, **78**, 183.
KLINKE, J.—(1940) *Klin. Wschr.*, **19**, 585.
KNOX, R.—(1939*a*) *J. Path. Bact.*, **49**, 469.—(1939*b*) *Brit. J. exp. Path.*, **20**, 391.
MANN, IDA.—(1949*a*) *Brit. J. Cancer*, **3**, 255.—(1949*b*) *Brit. med. J.*, ii, 251.—(1949*c*) *Ibid.*, ii, 253.
MURPHY, J. B., AND LANDSTEINER, K.—(1925) *J. exp. Med.*, **41**, 807.
NAKAHARA, W.—(1926) *Gann*, **20**, 13.
*Idem* AND YAOI, H.—(1930), *Science*, **72**, 277
PASSEY, R. D., AND DMOCHOWSKI, L.—(1950) *Brit. med. J.*, ii, 1129.
*Iidem*, LASNITZKI, ILSE, AND MILLARD, A.—(1950) *Ibid.*, ii, 1134.
PIRIE, ANTOINETTE, AND HOLMES, BARBARA E.—(1931) *Brit. J. exp. Path.*, **12**, 127.
POLLARD, A.—(1939) *Ibid.*, **20**, 429.
REED, L. G., AND MUENCH, H.—(1938) *Amer. J. Hyg.*, **27**, 493.
ROUS, P.—(1913) *J. exp. Med.*, **18**, 416.
*Idem*, MCMASTER, P. D., AND HUDACK, S. S.—(1935) *Ibid.*, **61**, 657.
*Idem* AND MURPHY, J. B.—(1913) *Ibid.*, **17**, 219.—(1914) *Ibid.*, **19**, 52.
SELBIE, F. R., AND MCINTOSH, J.—(1939) *Brit. J. exp. Path.*, **20**, 443.
SIMONIN, C.—(1931) *C. R. Soc. Biol., Paris*, **107**, 1029.
SITTENFIELD, M. J., JOHNSON, B. A., AND JOBLING, J. W.—(1931*a*) *Proc. Soc. exp. Biol., N.Y.*, **28**, 517.—(1931*b*) *Amer. J. Cancer*, **15**, 2275.
TODD, C.—(1928) *Brit. J. exp. Path.*, **9**, 244.
WARNER, P. T. J. C. P., AND GOSTLING, J. V. T.—(1950) *Brit. J. Cancer*, **4**, 380.